# Topographic Variation in Aboveground Biomass in a Subtropical Evergreen Broad-Leaved Forest in China

**DOI:** 10.1371/journal.pone.0048244

**Published:** 2012-10-30

**Authors:** Dunmei Lin, Jiangshan Lai, Helene C. Muller-Landau, Xiangcheng Mi, Keping Ma

**Affiliations:** 1 State Key Laboratory of Vegetation and Environmental Change, Institute of Botany, Chinese Academy of Sciences, Beijing, China; 2 University of Chinese Academy of Sciences, Beijing, China; 3 Smithsonian Tropical Research Institute, Balboa, Ancon, Republic of Panama; University of Zurich, Switzerland

## Abstract

The subtropical forest biome occupies about 25% of China, with species diversity only next to tropical forests. Despite the recognized importance of subtropical forest in regional carbon storage and cycling, uncertainties remain regarding the carbon storage of subtropical forests, and few studies have quantified within-site variation of biomass, making it difficult to evaluate the role of these forests in the global and regional carbon cycles. Using data for a 24-ha census plot in east China, we quantify aboveground biomass, characterize its spatial variation among different habitats, and analyse species relative contribution to the total aboveground biomass of different habitats. The average aboveground biomass was 223.0 Mg ha^−1^ (bootstrapped 95% confidence intervals [217.6, 228.5]) and varied substantially among four topographically defined habitats, from 180.6 Mg ha^−1^ (bootstrapped 95% CI [167.1, 195.0]) in the upper ridge to 245.9 Mg ha^−1^ (bootstrapped 95% CI [238.3, 253.8]) in the lower ridge, with upper and lower valley intermediate. In consistent with our expectation, individual species contributed differently to the total aboveground biomass of different habitats, reflecting significant species habitat associations. Different species show differently in habitat preference in terms of biomass contribution. These patterns may be the consequences of ecological strategies difference among different species. Results from this study enhance our ability to evaluate the role of subtropical forests in the regional carbon cycle and provide valuable information to guide the protection and management of subtropical broad-leaved forest for carbon sequestration and carbon storage.

## Introduction

The carbon cycle of the earth has been massively altered by anthropogenic activities [1]–[3]. Forests represent a major reservoir of global carbon, and play a major role in regional and global carbon cycles because they act as carbon sinks during succession and as carbon sources when destroyed or degraded by human or natural disturbances [4], [5]. An accurate estimate of the magnitude of carbon stocks in forest ecosystems within different climatic regions is therefore essential for understanding global and regional carbon budgets [2]. As a result, there have been increased efforts during the past several decades to estimate carbon stocks in forest ecosystems [1], [6]–[10].

In forest ecosystems, carbon is stored mostly in two pools: biomass and soil organic matter. Much of the carbon in soil is physically and chemically kept and not easily oxidized [11], [12]. In contrast, biomass, particularly aboveground biomass, is vulnerable to natural and anthropogenic disturbances, such as wildfire, logging, land conversion, storms, and insect or disease outbreaks [4]. Thus, the dynamic of aboveground biomass will dominate the short-term response of carbon storage in forest ecosystems, and are appropriately the dominant focus of research on forest carbon pools.

Due to the influence of the Tibetan plateau, subtropical region of China lacks a dry belt [13]. Subtropical evergreen broad-leaved forests rich in biodiversity are thus widely distributed. These forests developed under subtropical monsoon climate characterized by hot, wet summers and slightly cold, dry winters and distinct four seasons [14], [15]. Annual mean rainfall of this region range from 1000 mm to 2000 mm (unevenly distributed among different months) and annual mean temperature range from 14°C to 22°C [14], [15]. Species of Fagaceae, Lauraceae and Theaceae dominate these forests and *Cyclobalanopsis*, *Castanopsis* and *Lithocarpus*, in general, are the dominants on the canopy [14], [15]. This forest type covers approximately a quarter of the area of China, therefore, possesses great social and ecological benefits [14]–[16]. Many studies have demonstrated that it plays a critical role in regional carbon storage and cycling [5], [6], [8], [17]. These forests appear unique in terms of their climate characteristics, forest structure and species composition [15], therefore, predictions of biomass based on data from temperate and tropical forests in the world may not yield reliable estimate for this region [9], [18]–[21]. In addition, large areas of the forests have been deforested or converted to agricultural land, and subsequently abandoned and naturally regenerate to secondary forests over the past several decades [16]. Old growth forests primarily survive in remote mountain ranges that are less accessible for land use. Commonly, old growth forests can be expected to provide a reasonable estimate of the upper limit of carbon storage for similar forest types [9], [22]–[24]. Therefore, better understanding the carbon storage of the old growth forests will provide valuable information for estimating carbon sequestration potential of the secondary forest and quantifying the amount of carbon lost as a result of past land-use activities. A number of field-based estimates of subtropical forests biomass have been carried out in China and some of them were conducted in old growth forests (Table S1). However, there is still large uncertainty regarding the aboveground biomass estimates for subtropical forests in China because most previous studies were based on measurements over extremely small areas (Table S1), and thus are subject to large sampling errors and may fail to adequately characterize the actual aboveground biomass of the study sites [25], [26]. Therefore, study of larger sampling area is needed to get a more accurate biomass value of subtropical old growth forests in China.

In addition, even for other forest types for which aboveground biomass is better known, there is limited information on local variation in biomass and determinants. What is the within-site variability of aboveground biomass, especially with respect to local variation in habitat? Do species contribute similarly or differently to the aboveground biomass of different habitats? These knowledge gaps in part reflect the fact that field measurements of biomass over large areas is expensive and time consuming [2], [27]. Nevertheless, these questions merit greater attention. Answering the first question will enhance our understanding of the impacts of disturbance – whether natural or anthropogenic – on the carbon cycle of China’s subtropical forest ecosystem, thus improving our ability to manage forested landscapes for carbon sequestration. Answering the second question can guide selection of species for regional scale reforestation, especially where the aim is to reforest for maximal carbon storage. For the second question, we expected that (i) individual species contribute differently to total aboveground biomass of different habitats due to species accumulate more biomass in the adapted habitats and less biomass in the unfavorable habitats, and (ii) different species show differently in habitat preference in terms of biomass contribution driven by the more or less difference in resource requirements among different species.

The Gutianshan 24-ha forest dynamic plot in east China, which supports old-growth typical subtropical evergreen broad-leaved forest, was established in 2005 [28], [29]. All trees ≥1 cm in diameter at breast height were mapped, measured and identified to species-level. In the present study, we use these reliable and spatially explicit inventory data to (1) quantify the aboveground biomass of the study plot, (2) characterize the variation in aboveground biomass among different habitats in the plot, and (3) test the hypothesis that individual species contribute differently to total aboveground biomass of different habitats, and different species show differently in habitat preference in terms of biomass contribution.

## Materials and Methods

### Ethics Statement

No specific permits were required for the described field studies in and outside of Gutianshan National Nature Reserve (GNNR). The nature reserve is owned and managed by the state and its government and the location including the site for our sampling are not privately-owned or protected in any way. So, specific permission for non-profit research is not required. The field studies did not involved in endangered or protected species in this area.

### Study Site and Data Collection

**Figure 1:**
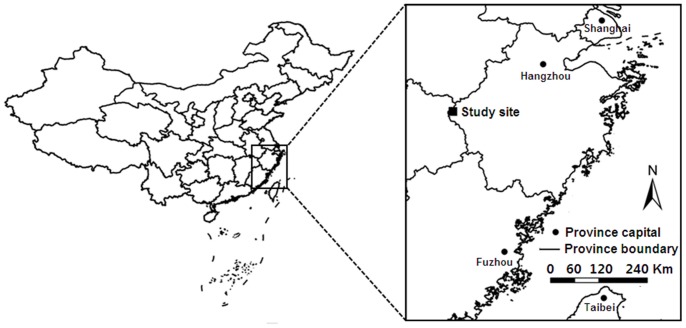
Location of the study site in Gutianshan National Nature Reserve, Zhejiang province, East China.

We conducted this study in a subtropical evergreen broad-leaved forest located in Gutianshan National Nature Reserve (GNNR), Zhejiang Province, East China (29°10′19″–29°17′41″ N, 118°03′50″–18°11′12″ E; Fig. 1). GNNR was established as a National Forest Reserve in 1975, and became a National Nature Reserve in 2001. The site is characterized by subtropical monsoon climate, with a mean annual temperature of 15.3°C and receives an average of 1964 mm of rain annually [30]. The topography is relatively undulating. The parent rock of the mountain range is granite, with soil pH ranging from 5.5 to 6.5 [31]. The predominant vegetation type of GNNR is subtropical evergreen broad-leaved forest [30]. A large portion of broad-leaved forests in the GNNR is in advanced successional stages [29] and a total of 1426 seed-plant species of 648 genera and 149 families have been recorded as occurring naturally in the GNNR, which covers 8107 ha in total [31].

**Figure 2:**
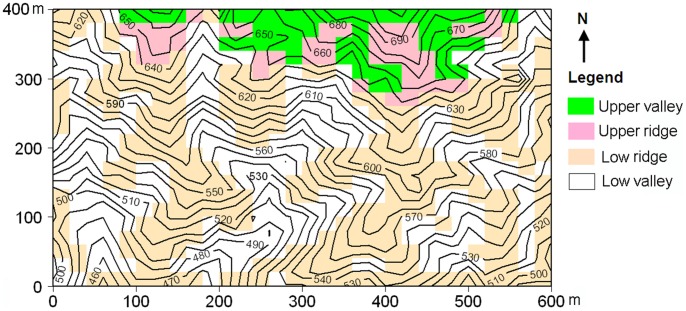
Topographic map of Gutianshan 24-ha plot with 10 m contour intervals. Each 20 m×20 m quadrat is categorized as one of four topographically defined habitats.

In December 2004, a 24-ha (600×400 m) permanent forest dynamic plot (hereafter Gutianshan plot) was established in the core area of the GNNR as part of the Chinese Forest Biodiversity Monitoring Network (CForBio; http://www.cfbiodiv.org/). Plot terrain is undulate with ridges and valleys; the elevation range from 446.3 m to 714.9 m, and 20-m cell slopes range from 13°to 62° (Fig. 2). In 2005, all trees ≥1 cm in diameter at breast height (1.3 m; dbh) were mapped, tagged, identified to the species-level, and had their diameters measured to the nearest millimeter (using dbh tapes). When a bole irregularity was present at 1.3 m, we measured 10 cm above the irregularity. We encountered 159 species belonging to 103 genera and 49 families in the plot. Common species in the plot include *Castanopsis eyrei* (Fagaceae), *Schima superba* (Theaceae), *Rhododendron ovatum* (Ericaceae), *Eurya muricata* (Theaceae), *Neolitsea aurata* var. *chekiangensis* (Lauraceae), *Chimonanthus salicifolius* (Calycanthaceae), *Camellia chekiang-oleosa* (Theaceae), *Daphniphyllum oldhamii* (Daphniphyllaceae), *Ternstroemia gymnanthera* (Theaceae), and *Loropetalum chinense* (Hamamelidaceae). For a more detailed description of the study plot and the tree species, the reader is referred to Chen et al. [28] and Legendre et al. [29].

### Aboveground Biomass Estimate

In this paper aboveground biomass was defined to include the aboveground oven-dried mass (stem, branches, and leaves) of living plants with dbh ≥1 cm. Total aboveground biomass was estimated using allometric equations (Table 1), and was expressed in Mg ha^−1^. For individuals with multiple stems, we calculated aboveground biomass of each stem and summed them.

**Table 1:** Allometric equations for aboveground biomass used in this study.

Species	Allometric equations	R^2^
*Schima superba*	*AGB* = 0.07103×(*D* ^2^×*H*)^0.91^	0.96
*Pinus massoniana*	*AGB* = 0.1359×(*D* ^2^×*H*)^0.79^	0.91
*Cyclobalanopsis glauca*	*AGB* = 0.08542×(*D* ^2^×*H*)^0.91^	0.93
*Castanopsis carlesii*	*AGB = *0.0453×*D* ^1.716^+0.037×*D* ^2.4599^+0.1565×*D* ^2.2772^	>0.98
*Cyclobalanopsis myrsinaefolia*	*AGB = *0.1019×exp(0.1387×*D*)+0.0358×*D* ^2.4556^+0.3152×*D* ^2.016^	>0.96
*Castanopsis eyrei*	*AGB* = 0.06491×(*D* ^2^×*H*)^0.92^	0.98
*Lithocarpus glaber*	*AGB* = 0.04268×(*D* ^2^×*H*)^0.98^	0.99
*Quercus* spp.	*AGB* = 0.1199×(*D* ^2^×*H*)^0.8509^	0.99
*Alniphyllum fortunei*	*AGB = *0.8003×(*D* ^2^×*H*)^0.5276^+0.1768×(*D* ^2^×*H*)^0.5648^+0.564×(*D* ^2^×*H*)^0.3191^	>0.95
*Loropetalum chinense*	*AGB = *0.1599×*D* ^2.35119^	0.99
*Rhododendron ovatum*	*AGB = *0.3323×*D* ^1.7874^	0.96
*Rhododendron latoucheae*	*AGB = *0.2212×*D* ^1.9932^	0.92
Other species	*AGB = *0.09459× (*D* ^2^×*H*)^0.87^	0.91

*AGB*: aboveground dry biomass (kg), *D*: diameter at breast height (cm), *H*: tree height (m).

Previously published species-specific allometric equations were available for C. eyrei [32], S. superba [8], Pinus massoniana [8], Cyclobalanopsis glauca [8], Cyclobalanopsis myrsinaefolia [10], Castanopsis carlesii [10], Lithocarpus glaber [32], and Alniphyllum fortunei [33], and a genus-specific allometric equation was available for Quercus serrata var brevipetiolata [34]. Where multiple equations were available for the same species, we used the one developed for the closest geographic location [27]. For R. ovatum, Rhododendron latoucheae, and L. chinense, we created species-specific allometric equations based on destructively sampled trees that span the range of diameters present in our study plot. We performed destructive sampling of 7 trees of R. ovatum, 7 trees of R. latoucheae, and 12 trees of L. chinense (all outside the Nature Reserve). After trees were harvested, each tree was separated into stem, branches, and leaves, and these fractions were separately weighed (to determine the fresh weight) using a portable electronic balance with an accuracy of ±20 g. Sub-samples of each part were collected, and oven dried at 80°C to constant weight to determine the moisture content (%). We calculated the oven-dried biomass of each tree section as oven-dried weight = fresh weight× (1–moisture content). We regressed log-transformed oven-dried aboveground biomass against log-transformed dbh for each of the 3 species. Back-transformed aboveground biomass estimates were multiplied by the correction factor CF = e^(MSE/2)^, where MSE is the mean square error of the regression [35]. The resulting allometric equations had R^2^>0.92 for each species (Table 1; Fig. S1). Over 35% of the individuals, accounting for about 74% of the total basal area, are covered by species-specific allometric equations. We used a generic allometric equation to estimate aboveground biomass of the remaining species [8]. All equations used in this study were derived from study areas with similar climates.

Most allometric equations used in this study required information on tree height. We used allometric equations relating height to diameter to predict height in these cases. We developed these equations from measurements of tree heights on a size-stratified random sample of 1066 trees in Gutianshan plot, measurements made using a telescopic measuring pole for trees <10 m and a laser rangefinder and clinometer for taller trees. Height was modeled as a function of diameter using the linear model: ln(*H*) = ln (*a*) + *b*ln(*D*), where *H* is tree height, *D* is dbh, *a* and *b* are the species-specific regression coefficients [36]. Each linear model was then back-transformed to a power function of the form: *H* = *aD^b^*×*CF*, where *CF* is correction factor. We fitted 47 species-specific tree height equations, which encompassed all common species (Table S2). An equation based on combined data from all species was used for species lacking species-specific equations (Table S2).

### Habitat Classification and Forest Structure in Different Habitats

In a previous analysis of beta diversity in Gutianshan plot, Legendre et al. [29] divided the plot into 600 20×20 m quadrats and used a multivariate regression tree to assign each quadrat to one of five habitats containing similar topographic conditions. Four topographic attributes – elevation, convexity, slope, and aspect – were calculated for each 20×20 m quadrat using the methods described in Harms et al. [37]. A detailed description of the topographic parameters of the five habitats can be found in Table 1 in Lai et al. [38]. One of the five habitats had only eight quadrats and was spatially aggregated in the study plot. Thus, Lai et al. [38] merged this habitat with the most similar other habitat in his study. In this study, we used the four habitats of Lai et al. [38]: low valley, low ridge, upper valley and upper ridge (Fig. 2).

We use basal area density and tree density to describe forest structure of different habitats. Basal area was calculated by summing the cross-sectional area of each stem at breast height, including the secondary stems that branch from the main stem below breast height.

### Relative Contributions of Species to Aboveground Biomass in Different Habitats

We focused our analysis of the relative contributions of different species to biomass on those species that ranked in the top 10 in aboveground biomass in one or more habitats. For each of these species, we calculated the relative contribution to total aboveground biomass of each habitat and of the entire plot.

We used a modification of the torus-translation randomization test described by Harms et al. [37] to test whether a given species contributed significantly more or less to one or more habitats (that is, whether the aboveground biomass distribution of a given species is significantly negatively or positively associated with one or more habitats). The torus-translation test takes into account the spatial autocorrelation inherent in both plant and habitat distributions by permuting rotations of habitat coordinates relative to those of trees [37], [38]. This method generates a null distribution of habitat occurrence for each focal species population, and then tests whether the observed relative contribution of each focal species on each habitat is significantly greater or lower than the random expectation. To obtain the expected values, the habitat map is overlayed on the tree distribution map, and translated while the tree distribution map remains fixed, and the edges of the habitat map wrap back on either side of the plot. At our study plot (consisting of 600 20×20 m quadrats), 599 unique torus translated habitat maps were initially possible (not including the true habitat map). From this, it is possible to generate three original maps to continue the two-dimensional torus translation: 180°rotation, mirror image and 180°rotation of the mirror image. In total, these procedures provide 2399 translated maps (not including the true habitat map), each of which provides a value of the expected relative contribution. The observed relative contribution of a species to a given habitat was compared to the frequency distribution of expected values. If the observed value was ≤2.5% or ≥97.5% of the expected values, then it was considered to have a significantly lower or higher contribution to the habitat at a significance level of 0.05. Similarly, if the observed value was ≤0.5% or ≥99.5%, then it was considered to have significantly lower or higher contribution to the habitat at a significance level of 0.01.

Values in this study are reported as means ±95% confident intervals. Bootstrap samples of 20×20 m quadrats were drawn 10,000 times with replacement to estimate 95% confident intervals. All analyses in this study were carried out using the software package R version 2.12 (R core team, 2010).

## Results

### Total Aboveground Biomass Estimate

**Figure 3:**
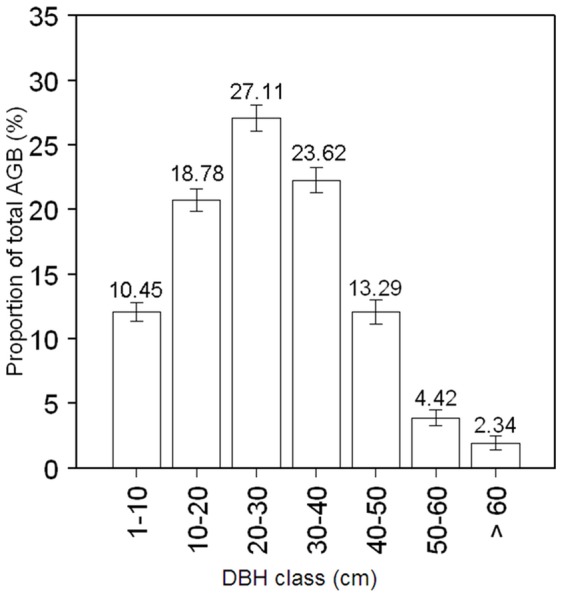
Distribution of tree aboveground biomass (AGB) among diameter classes (DBH class) in Gutianshan 24-ha plot. Error bars show 95% confidence intervals based on bootstrapping over 20×20 m quadrats.

Total aboveground biomass in the Gutianshan 24-ha plot was 223.0 Mg ha^−1^ (bootstrapped 95% CI [217.6, 228.5]; Table 2). Just 17 of 159 species contributed approximately 90% of the total aboveground biomass. The five species accounting for the largest proportion of aboveground biomass were *C. eyrei* (81.4 Mg ha^−1^), *S. superba* (50.1 Mg ha^−1^), *P. massoniana* (15.8 Mg ha^−1^), *Q. serrata* var *brevipetiolata* (9.3 Mg ha^−1^), and *C. glauca* (6.5 Mg ha^−1^). Trees 10–40 cm in diameter accounted for 69.5% of the aboveground biomass, while trees greater than 40 cm accounted for 20.1%, and trees less than 10 cm just 10.5% (Fig. 3).

**Table 2:** Summary of forest structure and aboveground biomass in four different habitats and entire plot of Gutianshan 24-ha plot.

Habitats	Area (ha)	Basal area (m^2^ ha^−1^)	Tree density (trees ha^−1^)	AGB (Mg ha^−1^)
Low valley	9.48	39.6 (38.2, 41.1)	7286 (7030, 7550)	209.9 (201.5, 218.3)
Low ridge	10.76	48.0 (46.6, 49.3)	7946 (7644, 8263)	245.9 (238.3, 253.8)
Upper valley	2.00	40.2 (37.4, 43.1)	12038 (10398, 13835)	199.4 (181.8, 218.3)
Upper ridge	1.76	40.6 (38.2, 43.1)	17664 (16108, 19203)	180.6 (167.1, 195.0)
Entire plot	24.00	43.5 (42.5, 44.4)	8739 (8405, 9081)	223.0 (217.6, 228.5)

Values are means with bootstrapped 95% confidence interval in parentheses. AGB: aboveground biomass.

### Habitat Differences in Stand Structure and Aboveground Biomass

**Figure 4:**
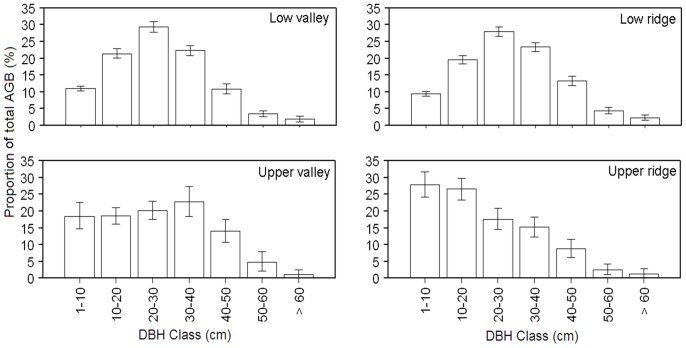
Distribution of tree aboveground biomass (AGB) among different diameter classes (DBH class) for the four habitats. Error bars show 95% confidence intervals based on bootstrapping over 20 m×20 m quadrats within each habitat type (i.e., if the habitat contains 100 20 m×20 m quadrats, 100 of these quadrats are sampled with replacement for each bootstrap iteration).

The four habitats differed in forest structure and aboveground biomass (Table 2; Fig. 4). Low valley had the lowest basal area (39.6 m^2^ ha^−1^, bootstrapped 95% CI [38.2, 41.1]), the lowest tree density (7286 trees ha^−1^, bootstrapped 95% CI [7030, 7550]), and slightly below average aboveground biomass. Low ridge had the largest basal area (48.0 m^2^ ha^−1^, bootstrapped 95% CI [46.6, 49.3]), the largest aboveground biomass (245.9 Mg ha^−1^, bootstrapped 95% CI [238.3, 253.8]), and low tree density (7946 trees ha^−1^, bootstrapped 95% CI [7644, 8263]), consistent with it having the largest number of big trees. The upper valley had intermediate basal area (40.2 m^2^ ha^−1^, bootstrapped 95% CI [37.4, 43.1]) and stem density (12038 trees ha^−1^, bootstrapped 95% CI [10398, 13835]), and somewhat below average aboveground biomass. The upper ridge contrasted sharply in forest structure from all other habitats: it had the highest stem density (17664 trees ha^−1^, bootstrapped 95% CI [16108, 19203]) and the lowest aboveground biomass (180.6 Mg ha^−1^, bootstrapped 95% CI [167.1, 195.0]), reflecting a high density of small trees.

### Habitat Differences in Major Contributors of Aboveground Biomass

**Figure 5:**
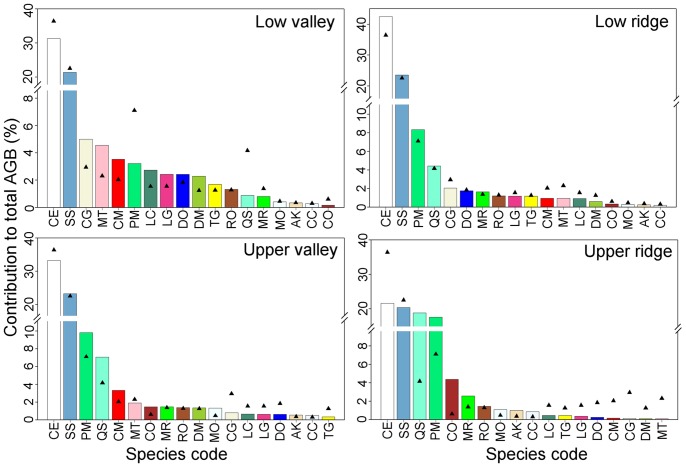
Relative contributions to total aboveground biomass (AGB) in different habitats for the 18 species that rank in the top 10 in one or more habitats. Species codes are defined in Table 3. Bars show relative contribution to aboveground biomass of the specific habitat while triangles show relative contribution to aboveground biomass of the entire plot; thus differences between bars and triangle represent the influence of species habitat associations. Aboveground biomass values of the 18 top-10 ranking species are given in Table S3.

We found that relatively few species dominated aboveground biomass in the entire plot and in individual habitats. The top-10 ranking species contributed 79–90% of the total aboveground biomass in each habitat (Fig. 5). The relative contribution of individual species differed significantly among different habitats (Table 3; Fig. 5). According to the torus-translation test, 15 of the 18 top-10 ranking species showed significant associations with one or more of the four habitats (Table 3). In total, 40 of 72 tests were significant at the individual test significance level of *P* = 0.05, and 28 were significant at *P* = 0.01(Table 3). Species show differently in habitat preference in terms of biomass contribution (Table 3; Fig. 5). Of the 15 species which show significant habitat associations, 13 species were positively associated with only one habitat but negatively associated with one or more of the other three habitats (Table 3). 8, 3, 0 and 6 species were positively associated with low valley, low ridge, upper valley and upper ridge, respectively (Table 3). Only *P. massoniana* and *Myrica rubra* were positively associated with both low ridge and upper ridge (Table 3).

**Table 3:** Relative contributions to total aboveground biomass in the four habitats of Gutianshan 24-ha plot for the 18 species that rank in the top 10 in one or more habitats, and tests for whether these contributions are significantly higher or lower in individual habitats by using torus-translation test.

	Low valley			Low ridge			Upper valley			Upper ridge		
Species (species code)	RC	*P*-value		RC	*P*-value		RC	*P*-value		RC	*P*-value	
*Castanopsis eyrei* (CE)	31.35	0.002	–	42.47	1.000	++	33.30	0.327		21.67	0.002	–
*Schima superba* (SS)	21.44	0.107		23.55	0.912		23.30	0.673		20.40	0.233	
*Pinus massoniana* (PM)	3.23	0.002	–	8.35	0.978	+	9.83	0.850		17.66	1.000	++
*Quercus serrata var. brevipetiolata* (QS)	0.89	0.002	–	4.42	0.675		7.09	0.813		18.87	1.000	++
*Cyclobalanopsis glauca* (CG)	5.01	1.000	++	2.06	0.002	–	0.81	0.023	−	0.15	0.002	–
*Machilus thunbergii* (MT)	4.56	1.000	++	0.95	0.001	–	1.91	0.380		0.09	0.002	–
*Cyclobalanopsis myrsinaefolia* (CM)	3.53	1.000	++	0.95	0.005	−	3.32	0.825		0.15	0.035	
*Daphniphyllum oldhamii* (DO)	2.42	0.998	++	1.75	0.297		0.63	0.020	−	0.24	0.002	–
*Lithocarpus glaber* (LG)	2.44	1.000	++	1.18	0.010	−	0.63	0.012	−	0.37	0.003	–
*Loropetalum chinense* (LC)	2.74	1.000	++	0.91	0.003	–	0.67	0.033		0.46	0.018	−
*Myrica rubra* (MR)	0.80	0.002	–	1.64	0.985	+	1.46	0.592		2.58	0.993	+
*Rhododendron ovatum* (RO)	1.33	0.717		1.22	0.143		1.37	0.708		1.43	0.733	
*Ternstroemia gymnanthera* (TG)	1.73	0.993	+	1.17	0.270		0.33	0.027		0.46	0.080	
*Distylium myricoides* (DM)	2.30	1.000	++	0.59	0.002	–	1.35	0.601		0.11	0.003	–
*Corylopsis glandulifera* var. *hypoglauca* (CO)	0.16	0.005	−	0.35	0.045		1.46	0.870		4.38	1.000	++
*Meliosma oldhamii* (MO)	0.42	0.395		0.27	0.037		1.33	0.935		1.10	0.898	
*Albizia kalkora* (AK)	0.34	0.447		0.25	0.007	−	0.54	0.838		1.00	0.998	++
*Camellia chekiang-oleosa* (CC)	0.28	0.381		0.20	0.002	–	0.52	0.830		0.86	0.997	++

RC: relative contribution (%). *P*-values are for the torus-translation tests, and are marked ‘+’ and ‘++’ for contributions that are significantly higher at individual test significance levels of 0.05 and 0.01, respectively and are marked ‘−’ and ‘−’ for contributions that are significantly lower at significance level of 0.05 and 0.01.

## Discussion

### Comparisons of Aboveground Biomass to Previous Studies

**Figure 6:**
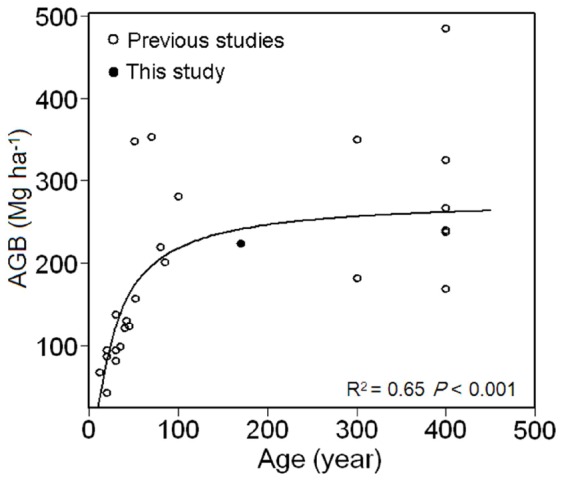
Relationship between forest stand age and forest aboveground biomass (AGB) of subtropical forests in China. Data is fitted by S-curve model y = e^(*a*+*b*/x)^ and data sources are listed in Table S1.

Estimated aboveground biomass measured in the plot and its habitats in this study are within the range of earlier reported values from other subtropical evergreen broad-leaved forests in China (Table S1). Considerable variation in aboveground biomass among subtropical evergreen broad-leaved forests in China is explained by forest stand age (Fig. 6). In addition, differences in climate [9], [18], [19], topography [39], [40], soil condition [39], [41], forest structure [20], and species composition [21] are also likely to contribute.

### Variation in Aboveground Biomass Among Habitats

Aboveground biomass varied substantially among the four topographic habitats in our study plot (Table 2). Low ridge supported the greatest aboveground biomass (245.9 Mg ha^−1^, bootstrapped 95% CI [238.3, 253.8]), whereas upper ridge held the lowest (180.6 Mg ha^−1^, bootstrapped 95% CI [167.1, 195.0]). High variation in aboveground biomass at local scales has been documented in other studies, and has been attributed to differences in topography, soil fertility, light condition, natural disturbance and their interaction [42]–[45].

Topography can influence the distribution of aboveground biomass in forests in multiple ways. Ferry et al. [44] found that steep slopes are associated with increased treefall mortality in a lowland rain forest in French Guiana. In a subtropical forest in Taiwan, high aboveground biomass was found in topographically flat areas [45]. In our study plot, the two valley habitats (low valley and upper valley) have many large rocks, perhaps because a small stream runs through them. Man et al. [46] found more trees uprooted after the ice storm disturbance in 2008 in the low valley habitat than in the other habitats in our study plot, and suggested this was due to the shallow soil and steep slopes. Besides the direct effects, terrain features greatly influence local conditions, especially soil processes, hydrology and light conditions [40], [42], which may in turn influence growth, mortality and recruitment, and thereby contribute to variation in aboveground biomass among habitats.

The positive relationship between soil nutrient availability and net primary productivity has been widely recognized, especially for soil nitrogen and phosphorus [47]–[49]. However, the relationship between soil fertility and aboveground biomass is less clear, as previous studies have yielded conflicting results [41], [42]–[44], [50]. In our study plot, the two valley habitats have higher soil fertility [51]. However, aboveground biomass in valley habitats is not higher than in the relative infertile habitat (low ridge; Table 2). Fertile soils should improve the stand productivity, leading to a more intense competition among trees [52]. Mortality rates may increase faster than growth rates on fertile soils, leading to lower biomass accumulation on fertile soils, at least in some cases [44], [53]. Further work is needed to investigate this possibility.

Light availability also varies with topography: it is higher on the ridges than in the valleys [42]. Consistent with this, many shade-tolerant species are found in valley habitats on our plot, while many light-demanding species are more abundant in the ridge habitats [24], [33]. For example, the most dominant light-demanding species, *C. eyrei*, contributes significantly more biomass to the total aboveground biomass of low ridge than that of low valley (one tailed *t*-test, t = −6.83, d*f* = 487.23, *P*<0.0001). These patterns suggest that light availability may be a major force in differences in species composition among habitats, and thereby perhaps also differences in aboveground biomass.

Finally, natural disturbances can also disrupt the carbon cycle of forest ecosystems and export carbon from the ecosystem [3], [54] and may have influenced patterns of aboveground biomass in our study site. Past disturbance may be the most important factor explaining the low aboveground biomass in upper ridge. This habitat was disturbed by fire, caused by local lightning strokes, in the 1960s [29]. The trees in this habitat are smaller and more numerous than in other habitats (Table 2), and include many individuals of pioneer or disturbance-related species, such as *Q. serrata* var. *brevipetiolata*, *Lyonia ovalifolia* var. *hebecarpa*, *Rhododendron mariesii*, *Albizia kalkor*, *Lindera reflexa*, and *Platycarya strobilacea* [29]. These patterns strongly suggest that this area is still recovering from the fire, and is at an earlier successional stage than the rest of the plot.

### Relative Contributions to Aboveground Biomass

In consistent with our expectation, our results clearly reveal that individual species contributed differently to the total aboveground biomass of different habitats, showing significant species habitat associations, and different species show differently in habitat preference in terms of biomass contribution (Table 3; Fig. 5). At least two different mechanisms might account for the patterns we detected. First, species differences in ecological strategies (niche differentiation) may account for the spatial segregation of species on different habitats [29], [37], [52], [55]. The complex topographic features of our study plot result in differential resource availability (soil nutrients, light and water) among different habitats [51]. Species are expected to accumulate higher biomass in areas with environmental conditions for which they are best adapted, thus result in the significant habitat associations in terms of biomass contribution. Second, seed dispersal limitation may also result in the spatial aggregation of specific species in specific habitat [56], thus result in the significant habitat associations in terms of biomass contribution. However, 13 of the 15 species, which show significant habitat associations in our test, were dispersal by rodents, birds and wind [57], suggesting that dispersal limitation is not severe at local scale [58]. Consequently, seed dispersal limitation is unlikely to account for the obvious patterns we detected.

Although biodiversity considerations have been taken into account only marginally in current international climate change mitigation initiatives [59], how can biodiversity affect forest carbon storage has captured much attention of scientists in the past few years [60]–[63]. Niche complementarity hypothesis has been proposed to explain the relationship between biodiversity and ecosystem function in many studies [60], [64]. This hypothesis predicts that more species take greater advantage of the niche opportunities that are available in an environment, thus result in more complete resource use [64]. In our study, we found that different species show differently in habitat preference in terms of biomass contribution (Fig. 5, Table 3), indicating that species may complement each other in space, thus keep high carbon storage at the whole plot scale. This may represent a form of niche complementarity [59], [65].

### Implications

Our study constitutes the largest (24-ha) whole-plot assessment of aboveground biomass in China to date (Table S1). Because the Gutianshan plot was specifically chosen to be representive of typical old-growth evergreen broad-leaved subtropical forest in this region, and because measurements in this plot were done very carefully and over a large area [29], we suggest that the aboveground biomass estimates presented here provide the best estimates to date of biomass of old-growth subtropical forests in this region. They thus constitute an important contribution to understanding the role of subtropical forests in the regional and global carbon cycle. However, because there is considerable spatial variation in aboveground biomass at large scales [9], [18], [20], [40], [41], measurements at more sites are required to determine the distribution of aboveground biomass for forests across the subtropical region of China as a whole. Our study also suggests that local scale biomass variations may be correlated with the local abiotic environment, and specifically topography. Therefore, when scaling up biomass estimates from stand to local or regional scales, inclusion of topographical and other environmental factors may substantially improve the accuracy of the larger-scale estimates.

Forest protection and reforestation are viable and low-cost strategies for mitigating rising levels of atmospheric CO_2_ and associated impacts of global climate change [66], [67]. Secondary forests have the potential to sequester a large amount of carbon as they regrow and approach old-growth stature [68], [69]. In Zhejiang province, evergreen broad-leaved forest covers roughly 263, 600 ha [70], with an average total biomass (aboveground and belowground biomass) of 89.19 Mg ha^−1^ [8]. If we assume that the below ground biomass is equal to 23.3% of aboveground biomass [71], then our measurements correspond to total biomass of 274.96 Mg ha^−1^. If we assume this is the carbon carrying capacity, namely the mass of carbon able to be stored in forest biomass under natural conditions without anthropogenic disturbance, about 24.49 Tg carbon could be accumulated by the growth of biomass in the evergreen broad-leaved forests of Zhejiang province (assumed 50% of biomass is carbon) over the coming decades if they were protected and allowed to naturally regenerate (thereby approaching this carrying capacity). Of course, the carbon sequestration potential of these forests varies over time and space, and not all may be capable of attaining this biomass density, but in any case, this does provide a perspective on the capacity of these secondary forests to mitigate CO_2_ emissions.

## Supporting Information

Figure S1 **Graphs of aboveground biomass against diameter and corresponding newly fitted allometric equations for** ***Rhododendron ovatum*** **,** ***Rhododendron latoucheae*** **, and** ***Loropetalum chinense*** **.**
(PDF)

Table S1 **Estimates of aboveground biomass in subtropical evergreen broad-leaved forests in China.**
(DOCX)

Table S2 **Tree height allometric equations for Gutianshan 24-ha plot.**
(DOCX)

Table S3 **Aboveground biomass of the 18 top-10 ranking species in Gutianshan plot.**
(DOCX)

## References

[1] Dixon RK, Brown S, Houghton RA, Solomon AM, Trexler MC, et al (1994) Carbon pools and flux of global forest ecosystems. Science 263: 185–190. 17839174 10.1126/science.263.5144.185

[2] Houghton RA (2005) Aboveground forest biomass and the global carbon balance. Global Change Biology 11: 945–958.

[3] Schulze ED (2006) Biological control of the terrestrial carbon sink. Biogeosciences 3: 147–166.

[4] Lorenz K, Lal R (2010) Carbon sequestration in forest ecosystems. Dordrecht: Springer.

[5] Pan Y, Birdsey RA, Fang J, Houghton R, Kauppi PE, et al (2011) A large and persistent carbon sink in the world’s forests. Science 333: 998–993. 10.1126/science.1201609 21764754

[6] Fang J, Wang G, Liu G, Xu S (1998) Forest biomass of China: An estimate based on the biomass-volume relationship. Ecological Applications 8: 1084–1091.

[7] Chave J, Condit R, Lao S, Casperesen JP, Foster RB, et al (2003) Spatial and temporal variation of biomass in a tropical forest: results form a large census plot in Panama. Journal of Ecology 91: 240–252.

[8] Zhang J, Ge Y, Chang J, Jiang B, Jiang H, et al (2007) Carbon storage by ecological service forests in Zhejiang Province, subtropical China. Forest Ecology and Management 245: 64–75.

[9] Keith H, Mackey B, Lindenmayer D (2009) Re-evaluation of forest biomass carbon stocks and lessons from the world’s most carbon-dense forests. Proceedings of the National Academy of Sciences of the United States of America 106: 11635–11640. 19553199 10.1073/pnas.0901970106 PMC2701447

[10] Yang T, Song K, Da L, Li X, Wu J (2010) The biomass and aboveground net primary productivity of *Schima superba*-*Castanopsis carlesii* forests in east China. Science in China (Series C: Life Sciences) 53: 811–821. 10.1007/s11427-010-4021-5 20697870

[11] Post WM, Kwon KC (2000) Soil carbon sequestration and land–use change: Processes and potential. Global Change Biology 6: 317–328.

[12] Davidson EA, Janssens IA (2006) Temperature sensitivity of soil carbon decomposition and feedbacks to climate change. Nature 440: 165–173. 16525463 10.1038/nature04514

[13] Kira T (1991) Forest ecosystems of east and southeast-Asia in a global perspective. Ecological Research 6: 185–200.

[14] Wu Z (1980) The Vegetation of China. Beijing: Science Press. [in Chinese].

[15] Zhong Z (1987) The typical subtropical evergreen broadleaved forest of China. Journal of Southwest China Normal University 3: 109–121.

[16] Wang X, Kent M, Fang X (2007) Evergreen broad-leaved forest in Eastern China: Its ecology and conservation and the importance of resprouting in forest restoration. Forest Ecology and Management 245: 76–87.

[17] Piao S, Fang J, Ciais P, Peylin P, Huang Y, et al (2009) The carbon balance of terrestrial ecosystems in China. Nature 458: 1009–1013. 19396142 10.1038/nature07944

[18] Malhi Y, Wood D, Baker TR, Wright J, Phillips OL, et al (2006) The regional variation of aboveground live biomass in old-growth Amazonian forests. Global Change Biology 12: 1107–1138.

[19] Stegen JC, Swenson NG, Enquist BJ, White EP, Phillips OL, et al (2011) Variation in aboveground forest biomass across broad climatic gradients. Global Ecology and Biogeography 20: 744–754.

[20] Baraloto C, Rabaud S, Molto Q, Blanc L, Fortunel C, et al (2011) Disentangling stand and environmental correlates of aboveground biomass in Amazonian forests. Global Change Biology 17: 2677–2688.

[21] Baker TR, Phillips OL, Malhi Y, Almeida S, Arroyo L, et al (2004) Variation in wood density determines spatial patterns in Amazonian forest biomass. Global Change Biology 10: 545–562.

[22] Smithwick EAH, Harmon ME, Remillard SM, Acker SA, Franklin JF (2002) Potential upper bounds of carbon stores in forests of the pacific northwest. Ecological Applications 12: 1303–1317.

[23] Keith H, Mackey B, Berry S, Lindenmayer D, Gibbons P (2010) Estimating carbon carrying capacity in natural forest ecosystems across heterogeneous landscapes: addressing sources of error. Global Change Biology 16: 2971–2989.

[24] Hoover C, Leak W, Keel B (2012) Benchmark carbon stocks from old-growh forests in northern New England, USA. Forest Ecology and Management 266: 108–114.

[25] Chave J, Condit R, Aguilar S, Hernandez A, Lao S, et al (2004) Error propagation and scaling for tropical forest biomass estimates. Philosophical Transactions of the Royal Society of London Series B-Biological Sciences 359: 409–420. 10.1098/rstb.2003.1425 PMC1693335 15212093

[26] Král K, Janík D, Vrska T, Adam D, Hort L, et al (2010) Local variability of stand structural features in beech dominated natural forests of Central Europe: Implications for sampling. Forest Ecology and Management 260: 2196–2203.

[27] Brown S (2002) Measuring carbon in forests: current status and future challenges. Environmental Pollution 116: 363–372. 11822714 10.1016/s0269-7491(01)00212-3

[28] Chen B, Mi X, Fang T, Chen L, Ren H, et al.. (2009) Gutianshan forest dynamic plot: Tree species and their distribution patterns. Beijing: China Forestry Publishing House.

[29] Legendre P, Mi X, Ren H, Ma K, Yu M, et al (2009) Partitioning beta diversity in a subtropical broad-leaved forest of China. Ecology 90: 663–674. 19341137 10.1890/07-1880.1

[30] Yu M, Hu Z, Yu J, Ding B, Fang T (2001) Forest vegetation types in Gutianshan National Natural Reserve in Zhejiang. Journal of Zhejiang University (Agriculture and Life Science) 27: 375–380. [in Chinese].

[31] Hu Z, Yu M, Suo F, Wu F, Liu Q (2008) Species diversity characteristics of coniferous broad-leaved forest in Gutian Moutain National Nature Reserve, Zhejiang province. Ecology and Environment 17: 1961–1964. [in Chinese].

[32] Du G, Hong L, Yao G (1987) Estimate and analysis the aboveground biomass of a secondary evergreen broad-leaved forest in Northwest of Zhejiang. Journal of Zhejiang Forestry Science and Technology 7: 5–12. [in Chinese].

[33] Chen W (2000) Study on the net productivity dynamic changes of the aboveground portion of Alniphyllum fortunei plantation. Journal of Fujian Forestry and Technology 27: 31–34. [in Chinese].

[34] Chen Q, Shen Q (1993) Studies on the biomass models of the tree stratum of secondary Cyclobalanopsis glauca forest in Zhejiang. Acta Phytoecologica Sinica 17: 38–47. [in Chinese].

[35] Sprugel DG (1983) Correcting for bias in log-transformed allometric equations. Ecology 64: 209–210.

[36] Köhl M, Magnussen SS, Marchetti M (2006) Sampling methods, remote sensing and GIS multiresource forest inventory. New York: Springer.

[37] Harms KE, Condit R, Hubbell SP, Foster RB (2001) Habitat associations of trees and shrubs in a 50-ha neotropical forest plot. Journal of Ecology 89: 947–959.

[38] Lai J, Mi X, Ren H, Ma K (2009) Species-habitat associations change in a subtropical forest of China. Journal of Vegetation Science 20: 415–423.

[39] de Castilho CV, Magnusson WE, de Araujo RNO, Luizao RCC, Lima AP, et al (2006) Variation in aboveground tree live biomass in a central Amazonian forest: Effects of soil and topography. Forest Ecology and Management 234: 85–96.

[40] Mascaro J, Asner GP, Muller-Landau HC, van Breugel M, Hall J, et al (2011) Controls over aboveground forest carbon density on Barro Colorado Island, Panama. Biogeosciences 8: 1615–1629.

[41] Laurance WF, Fearnside PM, Laurance SG, Delamonica P, Lovejoy TE, et al (1999) Relationship between soils and Amazon forest biomass: A landscape-scale study. Forest Ecology and Management 118: 127–138.

[42] Tateno R, Takeda H (2003) Forest structure and tree species distribution in relation to topography-mediated heterogeneity of soil nitrogen and light at the forest floor. Ecological Research 18: 559–571.

[43] Paoli GD, Curran LM, Slik JWF (2008) Soil nutrients affect spatial patterns of aboveground biomass and emergent tree density in southwestern Borneo. Oecologia 155: 287–299. 18038155 10.1007/s00442-007-0906-9

[44] Ferry B, Morneau F, Bontemps JD, Blanc L, Freycon V (2010) Higher treefall rates on slopes and waterlogged soils result in lower stand biomass and productivity in a tropical rain forest. Journal of Ecology 98: 106–116.

[45] McEwan RW, Lin Y, Sun I, Hsieh C, Su S, et al (2011) Topographic and biotic regulation of aboveground carbon storage in subtropical broad-leaved forests of Taiwan. Forest Ecology and Management 262: 1817–1825.

[46] Man X, Mi X, Ma K (2011) Effects of an ice strom on community structure of an evergreen broad-leaved forest in Gutianshan National Natural Reserve, Zhejiang Province. Biodiversity Science 19: 197–205. [in Chinese].

[47] Malhi Y, Baker TR, Phillips OL, Almeida S, Alvarez E, et al. 2004. The above-ground coarse wood productivity of 104 Neotropical forest plots. Global Change Biology 10: 563–591.

[48] Elser JJ, Bracken MES, Cleland EE, Gruner DS, Harpole WS, et al (2007) Global analysis of nitrogen and phosphorus limitation of primary producers in freshwater, marine and terrestrial ecosystems. Ecology Letters 10: 1135–1142. 17922835 10.1111/j.1461-0248.2007.01113.x

[49] Paoli G, Curran L (2007) Soil nutrients limit fine litter production and tree growth in mature lowland forest of southwestern Borneo. Ecosystems 10: 503–518.

[50] Schaik CPV (1985) Spatial variation in the structure and litterfall of a Sumatran rain forest. Biotropica 17: 196–205.

[51] Zhang L (2010) The effect of spatial heterogeneity of environmental factors on species distribution and community structure. Beijing, China: PhD thesis. Institute of Botany, Chinese Academy of Sciences. [in Chinese].

[52] Russo SE, Davies SJ, King DA, Tan S (2005) Soil-related performance variation and distributions of tree species in a Bornean rain forest. Journal of Ecology 93: 879–889.

[53] Pregitzer KS, Euskirchen ES (2004) Carbon cycling and storage in world forests: biome patterns related to forest age. Global Change Biology 10: 2052–2077.

[54] Running SW (2008) Climate change - Ecosystem disturbance, carbon, and climate. Science 321: 652–653. 18669853 10.1126/science.1159607

[55] Yamada T, Zuidema PA, Itoh A, Yamakura T, Ohkubo T, et al (2007) Strong habitat preference of a tropical rain forest tree does not imply large differences in population dynamics across habitats. Journal of Ecology 95: 332–342.

[56] Condit R, Ashton PS, Baker P, Bunyavejchewin S, Gunatilleke S, et al (2000) Spatial patterns in the distribution of tropical tree species. Science 288: 1414–1418. 10827950 10.1126/science.288.5470.1414

[57] Du YJ, Mi XC, Liu XJ, Ma KP (2012) The effects of ice storm on seed rain and seed limitation in an evergreen broad-leaved forest in east China. Acta Oecologica 39: 87–93.

[58] Vittoz P, Engler R (2007) Seed dispersal distances: a typology based on dispersal modes and plant traits. Botanica Helvetica 117: 109–124.

[59] Naeem S, Bunker DE, Hector A, Loreau M, Perrings C (2009) Biodiversity, Ecosystem Functioning, and Human Wellbeing. Oxford: Oxford University Press.

[60] Ruiz-Jaen MC, Potvin C (2010) Tree diversity explains variation in ecosystem function in a Neotropical forest in Panama. Biotropica 42: 638–646.

[61] Ruiz-Jaen MC, Potvin C (2011) Can we predict carbon stocks in tropical ecosystems from tree diversity? Comparing species and functional diversity in a plantation and a natural forest. New Phytologist 189: 978–987. 20958305 10.1111/j.1469-8137.2010.03501.x

[62] Vance-Chalcraft HD, Willig MR, Cox SB, Lugo AE, Scatena FN (2010) Relationship between aboveground biomass and multiple measures of biodiveristy in subtropical forest of Puerto Rico. Biotropica 42: 290–299.

[63] Caspersen JP, Pacala SW (2000) Successional diversity and forest ecosystem function. Ecological Research 16: 895–903.

[64] Cardinale BJ (2011) Biodiversity improves water quality through niche partitioning. Nature 472: 86–89. 21475199 10.1038/nature09904

[65] Loreau ML, Mouquet N, Gonzalez A (2003) Biodiversity as spatial insurance in hetegrogeneous landscapes. Proceedings of the National Academy of Sciences of the United States of America 100: 12765–12770. 14569008 10.1073/pnas.2235465100 PMC240692

[66] Canadell JG, Raupach MR (2008) Managing forests for climate change mitigation. Science 320: 1456–1457. 18556550 10.1126/science.1155458

[67] Imai N, Samejima H, Langner A, Ong RC, Kita S, et al (2009) Co-Benefits of sustainable forest management in biodiversity conservation and carbon sequestration. PLoS ONE 4: e8267. 20011516 10.1371/journal.pone.0008267 PMC2788241

[68] Hughes RF, Kauffman JB, Jarmillo V (1999) Biomass, carbon, and nutrient dynamics of secondary forests in a humid tropical region of Mexico. Ecology 80: 1892–1907.

[69] Shi L, Zhao S, Tang Z, Fang J (2011) The changes in China’s forests: An analysis using the forest identity. PLoS ONE 6: e20778. 21695254 10.1371/journal.pone.0020778 PMC3111435

[70] Liu A, Zhang Z, Ding Y (2002) The Natural Forest Resources of Zhejiang (the Volume of Forests). Beijing: Chinese Agriculture Science and Technology Press. [in Chinese].

[71] Fang J, Liu G, Xu S (1996) Biomass and net production of forest vegetation in China. Acta Ecologica Sinica 16: 497–508. [in Chinese].

