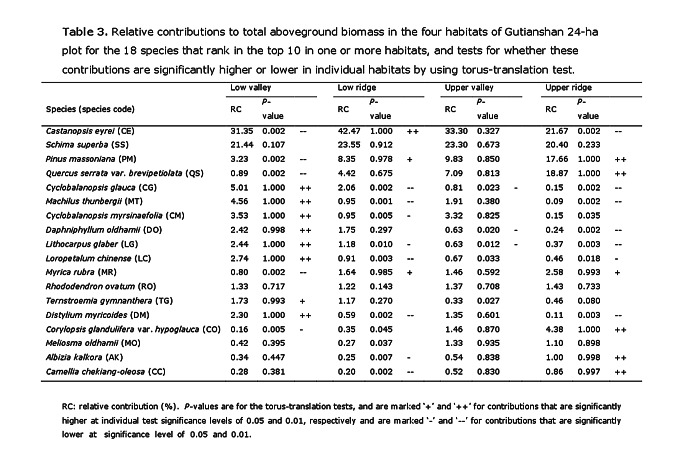# Correction: Topographic Variation in Aboveground Biomass in a Subtropical Evergreen Broad-Leaved Forest in China

**DOI:** 10.1371/annotation/779db3d7-8d77-4c40-8f64-c694c2e30912

**Published:** 2013-05-15

**Authors:** Dunmei Lin, Jiangshan Lai, Helene C. Muller-Landau, Xiangcheng Mi, Keping Ma

There were errors in the Funding statement and in Table 3.

The correct Funding statement is: This study was funded by National Natural Science Foundation of China (Grant No.31270496). The funder had no role in study design, data collection and analysis, decision to publish, or preparation of the manuscript.

A correct version of Table 3 is available here: 

**Figure:**