# Supplementary material for: Topographic Variation in Aboveground Biomass in a Subtropical Evergreen Broad-Leaved Forest in China
Source: PLoS One. 2012 Oct 30;7(10):e48244. doi: 10.1371/journal.pone.0048244 (PMC3484055; doi:10.1371/journal.pone.0048244)

Figure S1. The three species-specific biomass allometric equations newly fitted in this study: (a) *Rhododendron ovatum*; (b) *Rhododendron latoucheae*; (c) *Loropetalum chinense*.

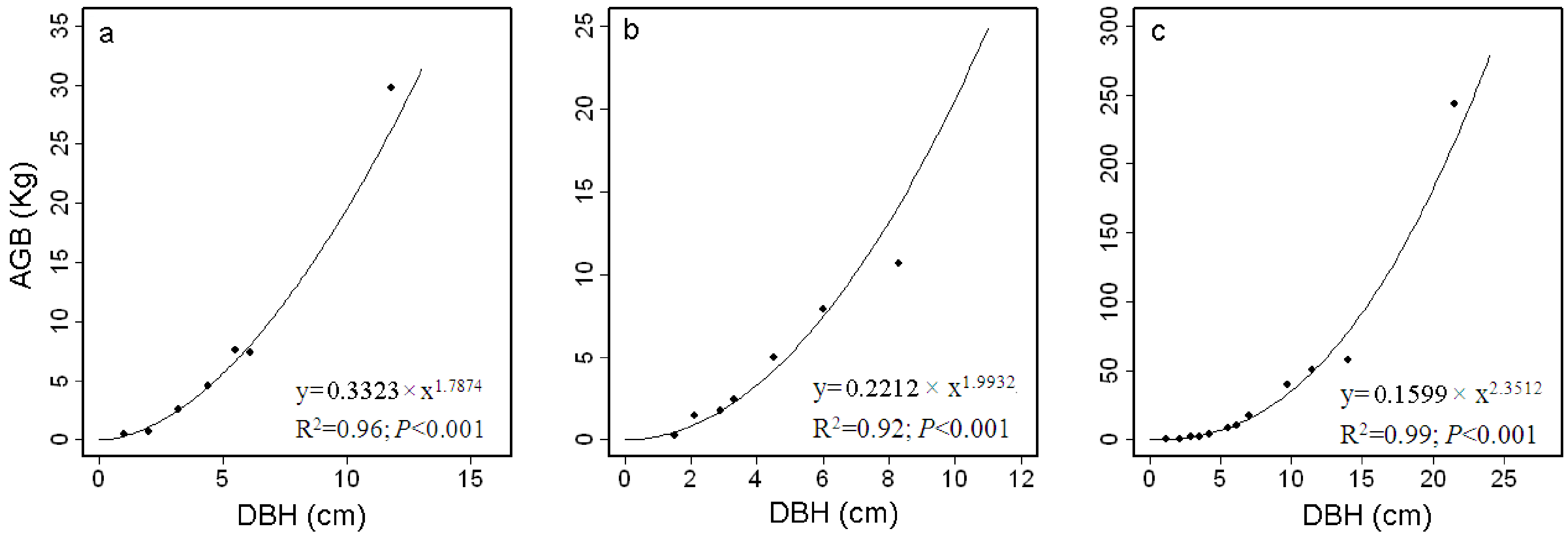

Supplement: Figure S1 — Graphs of aboveground biomass against diameter and corresponding newly fitted allometric equations for Rhododendron ovatum , Rhododendron latoucheae , and Loropetalum chinense . (PDF) [file pone.0048244.s001.pdf]
