# Supplementary material for: Topographic Variation in Aboveground Biomass in a Subtropical Evergreen Broad-Leaved Forest in China
Source: PLoS One. 2012 Oct 30;7(10):e48244. doi: 10.1371/journal.pone.0048244 (PMC3484055; doi:10.1371/journal.pone.0048244)
Supplement: Table S1 — Estimates of aboveground biomass in subtropical evergreen broad-leaved forests in China. (DOCX) [file pone.0048244.s002.docx]

Table S1. Estimates of aboveground biomass in subtropical evergreen broad-leaved forests in China. AGB: aboveground biomass. MAP: mean annual precipitation, MAT: mean annual temperature.

| **Study site** | **Longitude (°E)** | **Latitude (°N)** | **Age (yr)** | **MAP**  **(mm yr^-1^)** | **MAT (℃)** | **Sampling area (ha)** | **AGB (Mg ha^-1^)** | **Reference** |
| --- | --- | --- | --- | --- | --- | --- | --- | --- |
| Dinghushan, Guangdong | 112.53 | 23.18 | 400 | 1927 | 21 | - | 485 | [Yi et al., 2000](#_ENREF_16) |
| Dinghushan, Guangdong | 112.53 | 23.18 | 400 | 1927 | 21 | 0.04 | 324.8 | [Wen et al., 1999](#_ENREF_14) |
| Dinghushan, Guangdong | 112.53 | 23.18 | 400 | 1927 | 21 | 0.04 | 266.7 | [Wen et al., 1999](#_ENREF_14) |
| Dinghushan, Guangdong | 112.53 | 23.18 | 400 | 1927 | 21 | 1 | 239.8 | [Wen et al., 1997](#_ENREF_13) |
| Dinghushan, Guangdong | 112.53 | 23.18 | 400 | 1927 | 21 | 0.2 | 237.8 | [Peng and Zhang 1994](#_ENREF_12) |
| Dinghushan, Guangdong | 112.53 | 23.18 | 400 | 1927 | 21 | 0.26 | 169.2 | [Zhang and Ding 1996](#_ENREF_18) |
| Ailaoshan, Yunnan | 101.02 | 24.54 | 300 | 1931 | 11.3 | 0.25 | 350.14 | [Liu et al., 2002](#_ENREF_9) |
| Ailaoshan, Yunnan | 101.02 | 24.54 | 300 | 1931 | 11.3 | 0.25 | 181.79 | [Liu et al., 2002](#_ENREF_9) |
| Gutisnshan, Zhejiang | 118.12 | 29.25 | 170 | 1964 | 15.3 | 24 | 223.6 | This study |
| Heishiding, Guangdong | 111.32 | 23.45 | 100 | 1744 | 19.6 | - | 281.2 | [Chen 1993](#_ENREF_1) |
| Huangshan, Anhui | 118.15 | 30.13 | 85 | 1540 | 15.5 | 0.04 | 201 | [Yu 1996](#_ENREF_17) |
| Dinghushan, Guangdong | 112.53 | 23.18 | 80 | 1927 | 21 | 0.08 | 219.16 | [Fang et al., 2003](#_ENREF_6) |
| Huitong, Hunan | 109.26 | 26.67 | 70 | 1265 | 16.6 | 0.25 | 353.23 | [Deng et al., 2000](#_ENREF_4) |
| Tiantongshan, Zhejiang | 121. 78 | 29.8 | 52 | 1375 | 16.2 | 0.20 | 157.4 | [Yang et al., 2010](#_ENREF_15) |
| Wuyishan, Fujian | 117.68 | 27.7 | 51 | 2000 | 15 | 0.03 | 348 | [Lin et al., 1996](#_ENREF_8) |
| Baotianman, Henan | 111.88 | 33.47 | 45 | 900 | 15.2 | 0.04 | 123.5 | [Liu et al., 1998](#_ENREF_10) |
| Pu’e,Yunnan | 100.85 | 23.2 | 42 | 1250 | 19.4 | 0.2 | 130.26 | [Dang and Wu 1992](#_ENREF_2) |
| Jiande, Zhejiang | 119.52 | 29.4 | 40 | 1502 | 16.9 | 0.04 | 122 | [Yu 1996](#_ENREF_17) |
| Baotianman, Henan | 111.88 | 33.47 | 35 | 900 | 15.2 | 0.04 | 98.84 | [Liu et al., 2001](#_ENREF_11) |
| Jiande, Zhejiang | 119.52 | 29.4 | 30 | 1502 | 16.9 | 0.04 | 137.55 | [Du et al., 1987](#_ENREF_5) |
| Jiande, Zhejiang | 119.52 | 29.4 | 30 | 1502 | 16.9 | 0.04 | 94.67 | [Du et al., 1987](#_ENREF_5) |
| Jiande, Zhejiang | 119.52 | 29.4 | 30 | 1502 | 16.9 | 0.04 | 82.01 | [Du et al., 1987](#_ENREF_5) |
| Fuming, Yunnan | 102.55 | 25.32 | 20 | - | - | - | 94.32 | [Dang et al., 1994](#_ENREF_3) |
| Jiande, Zhejiang | 119.52 | 29.4 | 20 | 1502 | 16.9 | 0.04 | 86.8 | [Yu 1996](#_ENREF_17) |
| Qianyanzhou, Jiangxi | 114.5 | 26.67 | 20 | 1664 | 16.5 | 1.5 | 42.7 | [Li et al., 2007](#_ENREF_7) |
| Pu’e,Yunnan | 100.85 | 23.2 | 12 | 1250 | 19.4 | 0.07 | 67.13 | [Dang and Wu 1992](#_ENREF_2) |

**Reference**

Chen, Z., 1993. Studies on biomass and its allocation of the evergreen broad-leaved forest in Heishiding, Guangdong. Acta Phytoecologica Sinica 17, 289-298.[in Chinese]

Dang, C., Wu, Z., 1992. Studies on the biomass for *Castanopsis Echdnocarpa* community of monsoon evergreen broad-leaved forest. Journal of Yunnan University 14, 95-107. [in Chinese]

Dang, C., Wu, Z., Zhang, Z., 1994. Studies on the biomass of *Cyclobalanopsis delavayi* Community. Journal of Yunnan University 16, 205-209. [in Chinese]

Deng, S., Liao, L., Wang, S., Gao, H., Lin, B., 2000. Bioproductivity of *Castanopsis hysrix - Cyclobalanopsis glauca - Machilus pauhoi* community in Huitong, Hunan. Chinese Journal of Applied Ecology 11, 651-654. [in Chinese]

Du, G., Hong, L., Yao, G., 1987. Estimate and analysis the aboveground biomass of a secondary evergreen broad-leaved forest in Northwest of Zhejiang. Journal of Zhejiang Forestry Science and Technology 7, 5-12. [in Chinese]

Fang, Y., Mo, J., Peng, S., Li, D., 2003. Role of forest succession on carbon sequestration of forest ecosystems in lower subtropical China. Acta Ecologica Sinica 23, 1685-1694. [in Chinese]

Li, H., Wang, S., Gao, R., Yu, G., 2007. The carbon storage of the subtropical forest vegetation in central Jianxi Province. Acta Ecologica Sinica 27, 693-704. [in Chinese]

Lin, Y., Lin, P., Li, z., He, J., Liu, C., 1996. Biomass and Productivity of *Castanopsis eyrei* community in Wuyi Mountains. Journal of Xiamen University (Natural Science) 35, 269-275. [in Chinese]

Liu, W., Fox, J.E.D., Xu, Z., 2002. Biomass and nutrient accumulation in montane evergreen broad-leaved forest (*Lithocarpus xylocarpus* type) in Ailao Moutains, SW China. Forest Ecology and Management 158, 223-235.

Liu, Y., Wu, M., Guo, Z., Jiang, Y., Liu, S., Wang, Z., Liu, B., Zhu, X., 1998. Biomass and net productivity of *Quercus variabilis* forest in Baotianman Natural Reserve. Chinese Journal of Applied Ecology 9, 569-574. [in Chinese]

Liu, Y., Wu, M., Guo, Z., Jiang, Y., Liu, S., Wang, Z., Liu, B., Zhu, X., 2001. Studies on biomass and net production of *Quesercus acutidentata* forest in Baotianman Nature Reserve. Acta Ecologica Sinica 21, 1451-1456. [in Chinese]

Peng, S., Zhang, Z., 1994. Biomass, productivity and efficiency of radiation utilization of a typical vegetation in Dinghushan. Science In China (Series B) 24, 497-502. [in Chinese]

Wen, D., Wei, P., Kong, G., Zhang, Q., Huang, Z., 1997. Biomass study of the community of *Castanopsis chinensis, Cryptocarya concinna, Schima superb* in a southern China Reserve. Acta Ecologica Sinica 17, 497-504. [in Chinese]

Wen, D., Wei, P., Zhang, Q., Kong, G., 1999. Studies on biomass of the lower subtropical evergreen broad-leaved forests in a MAB Reserve of south China. Acta Phytoecologica Sinica 23, 11-21. [in Chinese]

Yang, T., Song, K., Da, L., Li, X., Wu, J., 2010. The biomass and aboveground net primary productivity of *Schima superba*-*Castanopsis carlesii* forests in east China. Science in China (Series C: Life Sciences) 53, 811-821.

Yi, W., Zhang, Z., Ding, M., Wang, B., 2000. Biomass and efficiency of radiation untilization in *Erythrophleum fordii* community. Acta Ecologica Sinica 20, 397-403. [in Chinese]

Yu, M., 1996. Dynamics of an evergreen broad-leaved forest dominated by *Cyclobalanopsis glauca* in southeast China. Scientia Silvae Sinicae 35, 42-51. [in Chinese]

Zhang, Z., Ding, M., 1996. Biomass and efficiency of radiation utilization in monsoon evergreen broadleaved forest in Dinghushan Biosphere Reserve. Acta Ecologica Sinica 16, 525-534. [in Chinese]
