# Supplementary material for: Topographic Variation in Aboveground Biomass in a Subtropical Evergreen Broad-Leaved Forest in China
Source: PLoS One. 2012 Oct 30;7(10):e48244. doi: 10.1371/journal.pone.0048244 (PMC3484055; doi:10.1371/journal.pone.0048244)
Supplement: Table S2 — Tree height allometric equations for Gutianshan 24-ha plot. (DOCX) [file pone.0048244.s003.docx]

Table S2. Parameters of a power function equation relating tree height (*H*, m) and diameter at breast height (*D*, cm) for the common species in Gutianshan 24-ha plot. The equation was *H* = *aD^b^*^,^ and was fitted through linear regression on log-transformed data. All parameters of all equations are significant at P < 0.001. *CF* is correction factor. The final model for height estimate is H = *CF*×*a*D*^b^*. Life forms include canopy trees (T), understory trees (U) and shrubs (S).

| **Family** | **Species** | **Chinese name** | **Life form** | ***a*** | ***b*** | **Adj.r^2^** | **min *D*** | **max *D*** | ***CF*** | **sample size** |
| --- | --- | --- | --- | --- | --- | --- | --- | --- | --- | --- |
| Pinaceae | *Pinus massoniana* | 马尾松 | T | 2.31514 | 0.56458 | 0.9118 | 3.7 | 80 | 1.010182 | 44 |
| Myricaceae | *Myrica rubra* | 杨梅 | T | 1.914162 | 0.54332 | 0.9466 | 1.9 | 41 | 1.007604 | 18 |
| Fagaceae | *Castanopsis carlesii* | 米槠 | T | 2.56075 | 0.5631 | 0.9232 | 1.3 | 42.5 | 1.015439 | 18 |
| Fagaceae | *Castanopsis eyrei* | 甜槠 | T | 2.240076 | 0.53683 | 0.9153 | 1.4 | 68.5 | 1.014401 | 58 |
| Fagaceae | *Castanopsis fargesii* | 栲树 | T | 1.714755 | 0.67197 | 0.9355 | 1.3 | 50.9 | 1.014428 | 22 |
| Fagaceae | *Castanopsis tibetana* | 钩栲 | T | 1.888325 | 0.6062 | 0.9457 | 1.5 | 43 | 1.01512 | 10 |
| Fagaceae | *Cyclobalanopsis glauca* | 青冈 | T | 2.31646 | 0.56941 | 0.933 | 1.2 | 37.9 | 1.015987 | 17 |
| Fagaceae | *Cyclobalanopsis myrsinaefolia* | 细叶青冈 | T | 3.068657 | 0.47664 | 0.9466 | 1.8 | 67.8 | 1.009587 | 13 |
| Fagaceae | *Lithocarpus glaber* | 石栎 | T | 2.420659 | 0.60426 | 0.9242 | 1 | 22.9 | 1.014613 | 20 |
| Fagaceae | *Quercus serrata var. brevipetiolata* | 短柄枹 | T | 2.566133 | 0.51193 | 0.8361 | 1.6 | 31.9 | 1.012184 | 52 |
| Magnoliaceae | *Michelia skinnneriana* | 野含笑 | T | 1.97157 | 0.60472 | 0.9377 | 1.5 | 18.7 | 1.004472 | 21 |
| Calycanthaceae | *Chimonanthus salicifolius* | 柳叶蜡梅 | S | 2.664216 | 0.55184 | 0.931 | 1.1 | 4.9 | 1.002477 | 12 |
| Lauraceae | *Cinnamomum chekiangense* | 浙江樟 | T | 1.711329 | 0.83537 | 0.9787 | 1.6 | 13.5 | 1.004013 | 10 |
| Lauraceae | *Cinnamomum subavenium* | 香桂 | T | 2.153146 | 0.63481 | 0.9464 | 1.3 | 24.5 | 1.010018 | 19 |
| Lauraceae | *Litsea coreana* var. *sinensis* | 豹皮樟 | T | 2.85628 | 0.55018 | 0.9417 | 1.5 | 20.3 | 1.005465 | 11 |
| Lauraceae | *Machilus grijsii* | 黄桢楠 | U | 2.41406 | 0.54758 | 0.8517 | 1.8 | 8.2 | 1.00806 | 7 |
| Lauraceae | *Machilus thunbergii* | 红楠 | T | 1.858835 | 0.67325 | 0.9425 | 2.4 | 34.5 | 1.01258 | 14 |
| Lauraceae | *Neolitsea aurata* var. *chekiangensis* | 浙江新木姜子 | U | 2.158298 | 0.69026 | 0.9558 | 1.1 | 14.4 | 1.006635 | 25 |
| Hamamelidaceae | *Corylopsis glandulifera var. hypoglauca* | 灰白蜡瓣花 | U | 2.265714 | 0.6837 | 0.9213 | 1.1 | 7.4 | 1.0049535 | 18 |
| Hamamelidaceae | *Distylium myricoides* | 杨梅叶蚊母树 | T | 2.632937 | 0.5521 | 0.9003 | 1 | 25.5 | 1.014236 | 28 |
| Hamamelidaceae | *Loropetalum chinense* | 檵木 | U | 3.038093 | 0.48234 | 0.8905 | 1.1 | 21.5 | 1.006925 | 24 |
| Rosaceae | *Sorbus folgneri* | 石灰花楸 | T | 3.149362 | 0.4941 | 0.9209 | 1.5 | 22 | 1.008834 | 15 |
| Daphniphyllaceae | *Daphniphyllum oldhamii* | 虎皮楠 | T | 2.267913 | 0.58382 | 0.9195 | 1.1 | 30.2 | 1.01025 | 20 |
| Anacardiaceae | *Toxicodendron succedaneum* | 野漆树 | T | 2.627282 | 0.56553 | 0.9444 | 1.7 | 35.3 | 1.008738 | 11 |
| Aquifoliaceae | *Ilex dlmerrilliana* | 榕叶冬青 | T | 2.243192 | 0.5951 | 0.9801 | 1.7 | 15.8 | 1.003303 | 6 |
| Aquifoliaceae | *Ilex litseaefolia* | 木姜叶冬青 | U | 2.398587 | 0.51503 | 0.9105 | 1.7 | 14 | 1.006217 | 15 |
| Aquifoliaceae | *Ilex chinensis* | 冬青 | T | 3.3253 | 0.46954 | 0.8977 | 1.9 | 26.3 | 1.006158 | 12 |
| Aquifoliaceae | *Ilex rotunda* | 铁冬青 | T | 2.131146 | 0.61152 | 0.9559 | 1.3 | 19 | 1.004018 | 13 |
| Aceraceae | *Acer cordatum* | 紫果槭 | T | 3.084162 | 0.45554 | 0.9133 | 1 | 10.7 | 1.004765 | 12 |
| Sabiaceae | *Meliosma oldhamii* | 红枝柴 | T | 2.883544 | 0.52441 | 0.8364 | 1.2 | 40.7 | 1.015099 | 27 |
| Elaeocarpaceae | *Elaeocarpus decipiens* | 杜英 | T | 2.088947 | 0.61295 | 0.9593 | 1.1 | 27.4 | 1.00709 | 17 |
| Elaeocarpaceae | *Elaeocarpus japonicus* | 薯豆 | T | 2.236227 | 0.61798 | 0.872 | 1.9 | 23.5 | 1.018784 | 10 |
| Theaceae | *Adinandra millettii* | 黄瑞木 | T | 2.280307 | 0.56137 | 0.909 | 1.6 | 18.3 | 1.007145 | 21 |
| Theaceae | *Camellia fraterna* | 毛花连蕊茶 | S | 2.030943 | 0.66278 | 0.9036 | 1.1 | 7.1 | 1.009085 | 10 |
| Theaceae | *Cleyera japonica* | 红淡比 | U | 1.572128 | 0.69064 | 0.9472 | 1.6 | 21.7 | 1.00927 | 19 |
| Theaceae | *Eurya muricata* | 隔药柃 | U | 2.304284 | 0.59205 | 0.854 | 1.6 | 14.6 | 1.011274 | 39 |
| Theaceae | *Schima superba* | 木荷 | T | 2.271976 | 0.57008 | 0.9416 | 1.2 | 55.5 | 1.007927 | 63 |
| Theaceae | *Ternstroemia gymnanthera* | 厚皮香 | T | 1.972773 | 0.63868 | 0.9384 | 1.3 | 30.8 | 1.012066 | 26 |
| Myrtaceae | *Syzygium buxifolium* | 赤楠 | U | 2.41708 | 0.5279 | 0.9152 | 1.1 | 19.3 | 1.006852 | 23 |
| Ericaceae | *Lyonia ovalifolia* var*. hebecarpa* | 毛果南烛 | U | 2.368413 | 0.4898 | 0.8528 | 1.6 | 7.6 | 1.006769 | 7 |
| Ericaceae | *Rhododendron latoucheae* | 麂角杜鹃 | U | 1.98165 | 0.56991 | 0.8869 | 1.5 | 10.2 | 1.010166 | 12 |
| Ericaceae | *Rhododendron ovatum* | 马银花 | U | 2.183218 | 0.5757 | 0.9084 | 1 | 13.6 | 1.008847 | 26 |
| Ericaceae | *Vaccinium bracteatum* | 乌饭树 | U | 1.873147 | 0.67845 | 0.9421 | 1.4 | 11.6 | 1.004564 | 9 |
| Ericaceae | *Vaccinium mandarinorum* | 江南越桔 | U | 2.234506 | 0.62323 | 0.9264 | 1.5 | 4.3 | 1.001825 | 7 |
| Symplocaceae | *Symplocos stellaris* | 老鼠矢 | U | 2.217898 | 0.59648 | 0.9303 | 1.4 | 12.1 | 1.006267 | 16 |
| Styracaceae | *Alniphyllum fortunei* | 拟赤杨 | T | 2.750162 | 0.59079 | 0.9008 | 1.5 | 41.2 | 1.017559 | 15 |
| Styracaceae | *Styrax odoratissimus* | 郁香野茉莉 | U | 3.220092 | 0.45692 | 0.8936 | 1.9 | 16.3 | 1.004416 | 10 |
| - | Other species | - | - | 2.353706 | 0.564976 | 0.9148 | 1 | 80 | 1.015602 | 1066 |
