# Supplementary material for: Topographic Variation in Aboveground Biomass in a Subtropical Evergreen Broad-Leaved Forest in China
Source: PLoS One. 2012 Oct 30;7(10):e48244. doi: 10.1371/journal.pone.0048244 (PMC3484055; doi:10.1371/journal.pone.0048244)
Supplement: Table S3 — Aboveground biomass of the 18 top-10 ranking species in Gutianshan plot. (DOCX) [file pone.0048244.s004.docx]

Table S3. Aboveground biomass of the 18 top 10-ranking species in Gutianshan plot.

| **Species** | **Entire plot**  **(Mg ha^-1^)** | **Low valley**  **(Mg ha^-1^)** | **Low ridge**  **(Mg ha^-1^)** | **Upper valley**  **(Mg ha^-1^)** | **Upper ridge**  **(Mg ha^-1^)** |
| --- | --- | --- | --- | --- | --- |
| *Castanopsis eyrei* (CE) | 81.39 | 65.93 | 104.67 | 66.56 | 39.26 |
| *Schima superba* (SS) | 50.12 | 44.82 | 57.69 | 46.29 | 36.73 |
| *Pinus massoniana* (PM) | 15.81 | 6.76 | 20.47 | 19.54 | 31.81 |
| *Quercus serrata var. brevipetiolata* (QS) | 9.29 | 1.87 | 10.87 | 14.13 | 34.11 |
| *Cyclobalanopsis glauca* (CG) | 6.60 | 10.54 | 5.08 | 1.63 | 0.27 |
| *Machilus thunbergii* (MT) | 5.15 | 9.57 | 2.33 | 3.82 | 0.17 |
| *Cyclobalanopsis myrsinaefolia* (CM) | 4.50 | 7.33 | 2.32 | 6.55 | 0.27 |
| *Daphniphyllum oldhamii* (DO) | 4.07 | 5.08 | 4.29 | 1.25 | 0.44 |
| *Lithocarpus glaber* (LG) | 3.48 | 5.13 | 2.91 | 1.26 | 0.66 |
| *Loropetalum chinense* (LC) | 3.44 | 5.74 | 2.24 | 1.33 | 0.83 |
| *Myrica rubra* (MR) | 3.05 | 1.68 | 4.02 | 2.90 | 4.65 |
| *Rhododendron ovatum* (RO) | 2.95 | 2.86 | 3.08 | 2.84 | 2.66 |
| *Ternstroemia gymnanthera* (TG) | 2.82 | 3.57 | 2.88 | 0.66 | 0.83 |
| *Distylium myricoides* (DM) | 2.80 | 4.82 | 1.45 | 2.69 | 0.21 |
| *Corylopsis glandulifera* var. *hypoglauca* (CO) | 1.33 | 0.58 | 0.85 | 2.89 | 7.86 |
| *Meliosma oldhamii* (MO) | 1.02 | 0.88 | 0.68 | 2.65 | 1.99 |
| *Albizia kalkora* (AK) | 0.77 | 0.70 | 0.61 | 1.08 | 1.80 |
| *Camellia chekiang-oleosa* (CC) | 0.65 | 0.34 | 0.49 | 1.04 | 1.56 |
